# The development of face orienting mechanisms in infants at-risk for autism

**DOI:** 10.1016/j.bbr.2012.07.030

**Published:** 2013-08-15

**Authors:** Mayada Elsabbagh, Teodora Gliga, Andrew Pickles, Kristelle Hudry, Tony Charman, Mark H. Johnson, the BASIS Team

**Affiliations:** a Department of Psychiatry, McGill University, 1033 Pine Avenue West, Montreal, QC, H3A 1A1, Canada; b Centre for Brain and Cognitive Development, School of Psychology, Birkbeck College, University of London, Malet Street, London, WC1E 7HX, United Kingdom; c Institute of Psychiatry, King's College London, 16 De Crespigny Park, London, SE5 8AF, United Kingdom; d Olga Tennison Autism Research Centre, School of Psychological Science, La Trobe University, Melbourne, Australia; e Centre for Research in Autism and Education, Department of Psychology and Human Development, Institute of Education, 25 Woburn Square, London, WC1H OAA, United Kingdom

**Keywords:** Autism, Infancy, At-risk, Face-processing, Attention, Prospective study

## Abstract

A popular idea related to early brain development in autism is that a lack of attention to, or interest in, social stimuli early in life interferes with the emergence of social brain networks mediating the typical development of socio-communicative skills. Compelling as it is, this developmental account has proved difficult to verify empirically because autism is typically diagnosed in toddlerhood, after this process of brain specialization is well underway. Using a prospective study, we directly tested the integrity of social orienting mechanisms in infants at-risk for autism by virtue of having an older diagnosed sibling. Contrary to previous accounts, infants who later develop autism exhibit a clear orienting response to faces that are embedded within an array of distractors. Nevertheless, infants at-risk for autism as a group, and irrespective of their subsequent outcomes, had a greater tendency to select and sustain attention to faces. This pattern suggests that interactions among multiple social and attentional brain systems over the first two years give rise to variable pathways in infants at-risk.

## Highlights

► Infants preferentially orient to socially relevant information such as faces. ► Infants at-risk for autism have a tendency to sustain attention to faces. ► Those infants who later develop autism show an equally strong face orienting response. ► Combined influence of social and attentional brain systems is implicated in autism.

## 1 Introduction

In typical adults, social stimuli and contexts are processed by specialized neural systems including cortical and sub-cortical structures [2,41]. Further specialization within this “social brain” network has been described. For instance, parts of the fusiform cortex appear to be involved in detecting and identifying faces [31]. Sub-cortical structures like the superior colliculus and the amygdala play a role in orienting to faces and to relevant facial information (e.g. eyes) [3]. The orbitofrontal cortex has been associated with encoding the reward value of social stimuli [5]. The developmental basis of these patterns of cortical specialization remains the subject of debate [52,32]. One proposed model suggests that this cortical specialization for faces is partly a result of early biases to orient toward and attend to faces. Specifically, [37] proposed that a subcortical orienting system (which they termed “Conspec”) initially biases the newborn to attend towards faces. This putative orienting system is driven by low-spatial frequency patterns characteristic of faces, and is sufficient to bias the input to developing cortical visual areas [38,39]. As a result of this biased input, alongside other constraints, over development some visual cortical areas become increasingly tuned to faces and related social stimuli. A manifestation of this functional specialization is the emergence of cortical tissue selectively activated by faces [40]. Based on this account we expect that infants’ face processing abilities will be characterized by both very early biases and experience-dependent developmental changes.

Partly motivated by such accounts of the emergence of the social brain in typical development, several groups have proposed that a lack of attention to, or interest in, social stimuli early in life may interfere with the emergence of developmental milestones that are critical for social learning, such as shared attention [13,20,38,39,60]. These cascading influences could preclude the typical development of socio-communicative, language and mentalizing skills, culminating in the behavioural presentation that characterises autism. Compelling as they are, the key elements of such developmental accounts have proved difficult to verify empirically.

Because a confirmed diagnosis of autism can only be made from around three years of age, most findings regarding preference for, or orienting to, various social and non-social stimuli have primarily been based on studies with older children and adults diagnosed with autism and have given rise to mixed findings. While some have suggested that face processing is the most informative model of the atypical development of the autistic brain [60], others have questioned whether difficulties in this area are universal [36]. Some of the inconsistencies have been attributed to possible changes over development in face orienting biases and face processing abilities (reviewed in [21]).

While no study has directly tested face orienting, several have documented difficulties in face processing in young children with autism, including recognition [10] and discrimination [12], as well as understanding of emotion [27] and eye gaze processing [9,38,39]. A few studies also documented atypical neural responses to faces in young children with autism [18,19,30]. These difficulties in childhood could be a result of reduced face expertise, in turn driven by an early impairment in face orienting. However, a few eye tracking studies draw a picture of emerging “disinterest” in faces during childhood. Assessment of children with autism at the age of two compared to those who are four-years-old suggests that relative to typically developing toddlers, toddlers with autism looked increasingly away from faces with age and attended atypically to key features of faces [10]. At four years of age, [1] also showed decreased attentional engagement to faces as measured by pupillary responses in children with autism relative to a control group. Atypical scanning and processing appear to be more pronounced in children relative to adults with autism, where findings are more mixed, suggesting the possibility that compensatory strategies may appear later in development (reviewed in [21,59]).

Notwithstanding these findings, other developmental models of autism have suggested that social orienting differences may not be the core deficits in autism, but instead that they originate from early general difficulties in controlling visual attention [6], which could, in turn, lead to problems in self-regulation as well as to a decrease in social orienting [43]. Because such deficits in visual attention are neither universal nor specific in autism, other researchers have proposed that an early specific deficit in orienting to socially relevant stimuli may be a necessary condition but probably not sufficient for autism to emerge. This deficit, however, would be compounded and amplified by the presence of visual attention difficulties [21]. Differences in social orienting would result in decreased input from socially relevant stimuli, while a problem with flexibly switching attention between different stimuli would result in ‘locking’ onto certain irrelevant aspects of the input (e.g. moving objects or, within the face, hairline instead of eyes). In support of this, one study which examined attentional disengagement from faces relative to objects found that toddlers with ASD disengaged visual attention from faces faster than developmentally delayed and typically developing toddlers [11]. These findings suggest that visual attention difficulties may also impair the acquisition of face processing skills.

As such, different hypotheses regarding the developmental origins and change in orienting to faces in autism are difficult to test in childhood once symptoms have become clearly expressed across multiple systems. Moreover, as described earlier, the human brain undergoes substantial development during the first years of life, with clear emergence and rapid change in social skill development in general and in face processing in particular. Indirect support for early differences in face orienting in autism come from retrospective studies looking back at the first two years of life using parental report or home videos. These studies show less orienting towards social stimuli and a reduced response to name calling from 9 months ([47,48]; Osterling et al., 2002; [64]) or younger [46] in children later diagnosed with autism, compared to those later diagnosed with developmental delay.

Against this background, a more recent approach has allowed for the prospective study of infants who are at increased risk for developing autism (for reviews see [24,66]). Later born siblings of children with autism are more likely to receive a diagnosis themselves as toddlers, relative to infants with no family history of autism. Interest in this group has been overwhelmingly driven by the search for ‘early markers’ as well as intermediate phenotypes, defined as autism-related characteristics observed in genetic relatives who do not have an autism diagnosis [24]. In other words, it is hoped that studying infant siblings may reveal the primary deficits in autism before symptoms are compounded by atypical interactions with the social and physical world, and before compensatory strategies and systems cloud the basic processing difficulties. Thus far, however, there has been little success in finding reliable markers for autism within the first year of life. On the one hand, infants below 12 months of age who are later diagnosed with autism show very few differences in the orienting to and scanning of faces when they interact with their caregiver [68] or with an experimenter [7,53]. By contrast, during the same period where infants at-risk show little behavioural difference from controls with no family history of autism [8,67], other studies using more direct measurements of brain activity have differentiated these groups in their response sensitivity to faces [22,23,50]. These early findings have motivated the view that understanding developmental changes in face orienting in infants at-risk as a group, and prior to the age of reliable diagnosis, will provide clues into variability in infants’ responses to genetic risk [24]. Moreover, because the majority of face orienting studies with infants at-risk have relied on observing behaviour within the context of complex interactions and in the absence of non-social stimuli, it remains possible that a more structured observational setting may reveal more sensitive indicators of social and communicative characteristics in toddlerhood.

In the current study we tested a group of infants at-risk for autism and a control group of infants with no family history for autism on a ‘face pop-out’ task [28]. The infants were administered the task twice, first around 7 months and again around 14 months of age. In this task, infants are presented with arrays of a face along with four non-social stimuli, including a ‘noise’ stimulus generated from the same face within the array created to match its low-level visual properties [33]. Previous findings using a similar task design showed a pronounced face preference in 6-months-olds across a range of eye tracking measures and stimulus presentation contexts in typically developing infants [28]. A first measure, the direction of the first look, singled out faces over other non-face objects (the face ‘pop-out’ effect) and was not affected by face inversion, with both upright and inverted faces attracting infant's first looks above what was expected by chance. When looking time was analysed, faces again received more fixations than other objects but infants also looked longer at upright than inverted faces. It was concluded that orienting is driven by more general face properties (e.g. the particular low spatial frequencies of the face), which may act through both sub-cortical and cortical mechanisms [38,39]. Once on the face, and having access to more visual detail, face-specific cortical mechanisms ensure that the more prototypical upright orientation maintains infants engaged with the face. Although initially designed to address questions about early social orienting, the complex displays used in this paradigm also allow us to examine measures of visual attention during early development.

In the current study, we derived a number of task-related eye tracking measures, to address three complementary questions regarding the origins of atypical development of the social brain in autism. The first question was whether infants who later go on to develop autism fail to selectively orient to faces. Consistent with previous studies [28], we operationalized this measure using the ‘pop-out’ response, i.e., above-chance proportion of first looks directed towards faces at the onset of each trial following a central fixation.

The second question was the extent to which the putative atypical development of general attention systems in infants at-risk [22] exerts an influence on visual selection in the current task. In other words, we tested whether automatic orienting to faces which typically occurs at the onset of a trial is followed by the optimal distribution of attention to other objects in the scene in the remainder of the trial. It is optimal for infants to both pay attention to social information, but also allocate some time to processing the other stimuli in the array, and an imbalance in any direction could be problematic. We operationalized the general allocation of attention using two measures: total looking time to the array and visual foraging, i.e., the number of Areas-of-Interest (AOIs) sampled. Because the pop-out response occurs early on in each trial (presentation of an array), and in view of previous findings highlighting time-dependent changes in visual scanning of scenes, we analysed separately the onset of each trial (first five seconds) and the remaining period [62,49].

The third question focused on the interaction between social orienting mechanisms and attention systems reflecting the integration of bottom-up and top-down processing mechanisms in the social brain. Such measures can only be ascertained indirectly. In the current study we derived face time, defined as the proportion of total looking time allocated to faces, and face foraging, defined as the likelihood that the face will be sampled relative to all other sampled AOIs. For consistency, we also analysed these measures separately for the trial onset and the remaining period.

## 2 Methods

### 2.1 Participants and clinical characterization

Recruitment, ethical approval (NHS NRES London REC 08/H0718/76) and informed consent, as well as background data on participating families, were made available for the current study through The British Autism Study of Infant Siblings (BASIS), a UK collaborative network facilitating research with infants at-risk for autism (www.basisnetwork.org/). Families enrol from various regions when their babies are younger than 5 months of age and they are invited to attend multiple research visits until their children reach three years of age or beyond. Each visit lasts a day or two and is adapted to meet the families’ needs. Measures collected are anonymised and shared among scientists to maximise collaborative value and to minimise burden on the families. A clinical advisory team of senior consultants works closely together with the research team/s and, if necessary, with the family's local health services, to ensure that any concerns about the child arising during the study are adequately addressed.

One hundred and four infants from BASIS took part in the current study (54 at-risk (21 male), and 50 low-risk (21 male). Along with several other measures, the infants were seen for the eye tracking task at the Centre for Brain and Cognitive Development when they were 6–10-months of age and again at 12–15 months. Subsequently, 52 (from 54) of those at-risk for ASD were seen for assessment around their second birthday (mean = 23.9 months, sd = 1.2) and 53 around their third birthday (mean = 37.7 months, sd = 3.0), by an independent team at the Centre for Research in Autism and Education, Institute of Education.

### 2.2 Confirmation of risk status in the older sibling

At the time of enrolment, none of the infants had been diagnosed with any medical or developmental condition. Infants at-risk all had an older sibling (hereafter, proband) with a community clinical diagnosis of ASD (or in 4 cases, a half-sibling), and in 3 cases 2 probands with an ASD. 45 probands were male, 9 were female. Proband diagnosis was confirmed by two expert clinicians (PB, TC) based on information using the Development and Wellbeing Assessment (DAWBA) [29] and the parent-report Social Communication Questionnaire (SCQ) [58]. Most probands met criteria for ASD on both the DAWBA and SCQ (*n* = 44). While a small number scored below threshold on the SCQ (*n* = 4) no exclusions were made, due to meeting threshold on the DAWBA and expert opinion. For 2 probands, data were only available for either the DAWBA (*n* = 1) or the SCQ (*n* = 1). For 4 probands, neither measure was available (aside from parent-confirmed local clinical ASD diagnosis at intake). Parent-reported family medical histories were examined for significant medical conditions in the proband or extended family members, with no exclusions made on this basis.

Infants in the low-risk group were recruited from a volunteer database at the Birkbeck Centre for Brain and Cognitive Development. Inclusion criteria included full-term birth (with one exception), normal birth weight, and lack of any ASD within first-degree family members (as confirmed through parent interview regarding family medical history). All low-risk infants had at least one older-sibling (in 3 cases, only half-sibling/s). 28 of the older siblings were male, 22 were female. Screening for possible ASD in these older siblings was undertaken using the SCQ, with no child scoring above instrument cut-off for ASD (≥15) (one score was missing).

### 2.3 Background characterisation measures

Two measures of general developmental level were obtained for the infants and toddlers at each visit. The Mullen Scales of Early Learning (MSEL) ([51]) is a direct assessment of verbal and non-verbal abilities appropriate for children from birth to 6 years. Scores across four domains – Visual Reception, Fine Motor, Receptive Language, and Expressive Language – are combined to yield an overall Early Learning Composite (ELC; mean = 100, sd = 15). Gross motor skills are also assessed but do not contribute to the ELC. An estimate of non-verbal developmental ability (NVT-score) was computed by averaging the T scores (mean = 50, sd = 10) for Visual Reception and Fine Motor subscales. The Vineland Adaptive Behaviour Scales (VABS) [61] is a parent-report measure of everyday skills in the domains of communication, daily living skills, social interaction, and motor skills. These combine to yield an adaptive behaviour composite (ABC; mean = 100, sd = 15).

These developmental assessments were undertaken at each of the visits, when infants were 6–10 months, 12–15 months, and again around the second and third birthday, each time by independent research teams. While the MSEL is always administered directly with the child, the VABS has alternative administration formats. The Parent/Caregiver Rating Form (i.e., questionnaire booklet) was used at the 6–10-month and 12–15-month visits, and the Survey Interview Form was used at the 24-month and 36-month visits. Scores from these measures are presented in Table 1.

### 2.4 Outcome characterization of the at-risk and low-risk groups

Alongside the standard measures of cognitive (MSEL) and adaptive (VABS) development taken at each visit, at 24 months (at-risk group only; 50 Module 1, 2 Module 2) and 36 months (both groups; Table 1, 3 Module 1, 98 Module 2) a semi-structured play assessment, the Autism Diagnostic Observation Schedule (ADOS) [44] was used to assess autism-related social and communication behavioural characteristics. This was augmented at 36 months (at-risk group only) with the parent-report Autism Diagnostic Interview – Revised (ADI-R) [45].

Characterisation of outcomes in the at-risk cohort at 36-months was done by ascertaining three sub-groups (Table 1): Those who were typically-developing, those classified as having ASD, and those exhibiting some form of developmental concerns. For the at-risk group consensus ICD-10 [65], ASD (including childhood autism; atypical autism, other pervasive developmental disorder (PDD)) was diagnosed using all available information from all visits by experienced researchers (TC, KH, SC, GP), hereafter At-risk-ASD. From the initial group of 53 toddlers assessed at 36-months, 17 (11 boys, 6 girls) met criteria for an ASD diagnosis (32.1%). Given the young age of the children, and in line with the proposed changes to DSM-5 [4], no attempt was made to assign specific sub-categories of PDD/ASD diagnosis. Another subgroup of toddlers from the at-risk group who were classified as not having ASD were considered to still have other developmental concerns. These were 12 toddlers (22.6%; 3 boys, 9 girls) who either scored above the ADOS or ADI [56] cut-off for ASD or scored <1.5SD on the Mullen ELC or RL and EL subscales but did not meet ICD-10 criteria for an ASD (9 scored > ADOS cut-off, 1 > ADOS cut-off and <1.5SD Mullen ELC cut-off, 1 > ADI cut-off, and 1 < 1.5SD Mullen ELC cut-off).

It is worth noting that the recurrence rate reported in the current study (32.1%) is higher than that reported in the large consortium paper recently published by Ozonoff and colleagues (18.7%). This is likely to reflect the modest size at-risk sample in the current study (*N* = 53). Whilst recurrence rates approaching 30% have been found in other moderate size samples (e.g., [42,55]) these rates are sample specific and will likely not be generalizable as findings from larger samples where autism recurrence rates converge on between 10% and 20% [16,54]. However, similar procedures combining all information from standard diagnostic measures and clinical observation and arriving at a ‘clinical best estimate’ ICD-10 diagnosis was used in the present study by an experience group to that used in other familial at-risk studies.

### 2.5 Face pop-out task at 6–10 months and 12–15 months: stimuli, procedure, and data processing

During their first and second visits infants were administered a face preference task very similar to that reported by [28]. Looking behaviour was recorded with a Tobii eye tracker. The Tobii system has an infrared light source and a camera mounted below a 17 in. flat-screen monitor to record corneal reflection data. The Tobii system measures the gaze direction of each eye separately and from these measurements evaluates where on the screen the individual is looking. During the eye tracker tasks the child is seated on his/her caregivers lap, at 50–55 centimeters from the Tobii screen. The height and distance of the screen are adjusted for each child to obtain good tracking of the eyes. First a five-point calibration sequence is run, with recording only started when at least four points are marked as properly calibrated for each eye. Gaze data were recorded at 50 Hz.

**Fig. 1:**
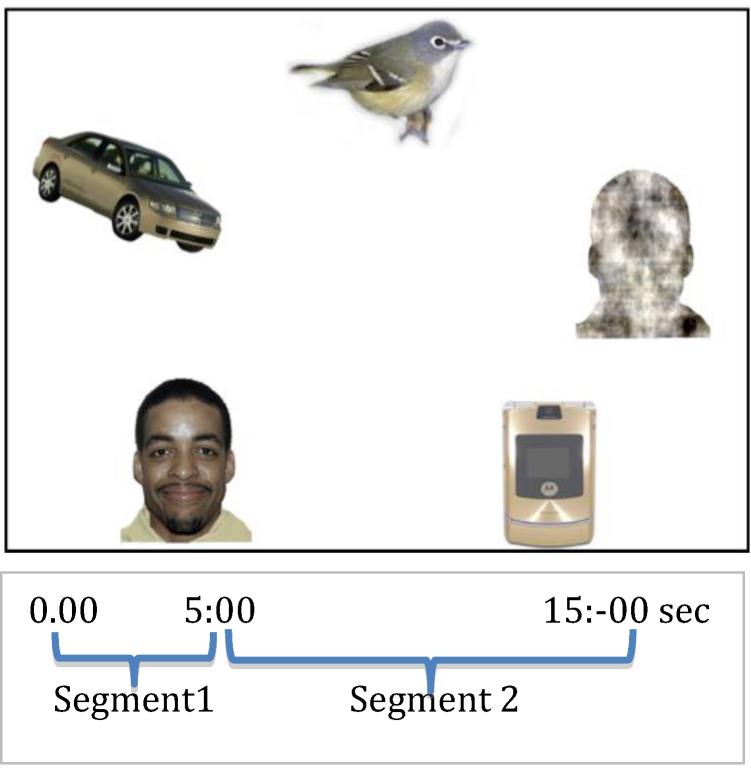
Example of stimuli and trial segmentation.

In the present task, 14 different arrays, each with five stimuli, were presented (see Fig. 1 for an example). Each array contained a colour image of one of fourteen different faces with direct gaze used as the target. Different exemplars from each of the following categories: mobile phones, birds, and cars were also included in the array. Another stimulus was a visual ‘noise’ image, generated from the same face presented within the array, by randomizing the phase spectra of the faces whilst keeping the amplitude and colour spectra constant [33]. The slides were counterbalanced for gender, ethnicity, and vertical and horizontal location of the face within the array. To verify that faces were similar to other categories in terms of visual saliency, saliency ranks were calculated for each area of interest on all 14 slides using the Saliency Toolbox 2.2 [63].^2^ Categories had very similar average saliency ranks. When placed at a distance of 55 cm from the child the five individual images on the slide had an eccentricity of 9.3° and covered an approximate area of 5.2° × 7.3°.

Before each slide a small animation was presented in the center of the screen to ensure that the children's gaze was directed to the centre. Each slide presentation lasted 15 s. To assist in maintaining the children's attention, the visual presentation was accompanied by music. If the child stopped looking at the slide one of the experimenters prompted the infant to look at the screen again, without naming or referring to any of the stimuli. When the infant looked away for more than 5 s, the experimenter terminated presentation of the given slide. Rectangular AOIs were defined around each object image and the center of the screen using Tobii Studio software. Gaze data were extracted for each AOI: centre, face, noise, car, bird, phone, and total (the entire slide).

#### 2.5.1 Eye tracking measures

##### 2.5.1.1 Face pop-out

Face pop-out response was tested using the proportion of valid trials where the infant was fixated at the centre at onset of the trial and then fixated one of the five AOIs corresponding to the face, against a chance level of .2. For this analysis, a trial was considered valid if the infant fixated the centre at onset of the trial and then moved their gaze to one of the five AOIs corresponding to any of the stimulus categories in the first three seconds of trial onset. Data were excluded for any infants with less than three valid trials. The measures are reported in Table 2.

##### 2.5.1.2 Total looking time

Total looking time was calculated as the total time spent looking at the array. Moreover, face looking time was calculated as the proportion of time spent on the face AOI relative to all target AOIs in the array. As discussed earlier, to highlight any temporal differences in these measures over the course of the trial, data were extracted for the first 5 s (first segment) of the trial time and for the remaining 10 s (final segment). Trials were considered valid if the infant spent longer than one second fixating the slide in total. Data were excluded for any infants with fewer than three valid trials. The measures are reported in Table 3.

##### 2.5.1.3 Visual foraging

Visual foraging was measured using the average number of AOIs sampled within the array over the course of each trial (ranging from 1 to 5). Face foraging was calculated as the ratio of face visiting (0 or 1) to the total AOIs visited. Similar to looking time measures, data was extracted for the onset and later segments of each trial. The same criteria for trial validity for looking time measures were applied to visual foraging. The measures are reported in Table 3.

## 3 Analytic approach

In addition to simple *t*-tests, the repeated measures data was analysed using a generalized estimating equations approach to fit the nature of proportion data. Infant first look behaviour within each of the two sets of trials (7-month and 14-month) was analysed as binomial proportions and logistic link function with robust standard errors to account for overdispersion and correlation between 7- and 14-month assessments. For the remaining measures, formed as averages over each segment of trials, of times, counts and their ratios, a Gaussian error, identity link, and an unstructured correlation matrix were used. Group differences were assessed from Wald tests with a parameter covariance matrix (and thus test statistics) calculated accounting for the number of parameters estimated. This approach is equivalent to multiple analysis of variance. Due to the variation of age within each group at the 7- and 14-months assessments stage, in all analyses the infant's age at was included as a covariate.

## 4 Results

### 4.1 Face pop-out

The proportion of valid trials in which the infant looked towards the face AOI before any other AOI was calculated for each group at each age (Table 2). One sample *t*-tests showed that the proportion of trials with first looks towards the face was significantly above chance level (.2) at 7-months for all groups defined based on risk status (control vs. at-risk) or on outcomes (at-risk: ASD, typical, other; all *p* < 0.001). Similar analyses were conducted for the second visit and the results did not differ. This demonstrates that the face pop-out effect was observed in all groups, including those with a clinical classification of ASD by the age of three years. Repeated measures analysis indicated no significant group differences in the rate of increase over the two assessments among the risk groups (group × time (*χ*^2^(1) = 0.23, *p* = .633) nor significant mean differences among the groups (group *χ*^2^(1) = 2.83, p = .092).

### 4.2 Total looking time and visual foraging measures

#### 4.2.1 Risk group effects

The average amount of looking anywhere in the array is shown in Table 3. A model was constructed with the following terms: between subjects groups (control, at-risk), and within-participants time (7-months vs. 14-months) and trial segment (first 5 s vs. last 10 s). In addition, age in months at the two points of measurement was used as a covariate. For the average time per trial examining AOIs the repeated measures analyses indicated no significant interactions involving group: group × time × segment (*χ*^2^(1) = 0⋅48, *p* = .490), group × segment (*χ*^2^(1) = 0⋅38, *p* = .535), group × time (*χ*^2^(1) = 1⋅79, p = .181).

For visual foraging, i.e., AOI count, the 3-way interaction of group × age × segment was not significant (*χ*^2^(1) = 0.06, *p* = .803) nor were the 2-way group × segment (*χ*^2^(1) = 0.06, *p* = .81) interactions but the group × time was marginal (*χ*^2^(1) = 3.28, *p* = .070) as was the group main effect (*χ*^2^(1) = 3.82, *p* = .051), with infants at-risk tending to sample fewer AOIs relative to the control group at the older age.

#### 4.2.2 Diagnostic group effects

The same analysis was repeated based on diagnostic classification of infants at 36-months (control, at-risk ASD, at-risk typical, at-risk other). No significant interactions involving diagnosis were observed: diagnosis × time × segment (*χ*^2^(3) = 3.10, *p* = .38), diagnosis × segment (*c*^2^(3) = 1.05, *p* = .79) diagnosis × time (*χ*^2^(3) = 6.2, *p* = .10). For visual foraging, i.e., AOI count, the 3-way interaction of diagnosis × age × segment was not significant (*χ*^2^(3) = 3.0, *p* = .39) nor were the 2-way diagnosis × time (*χ*^2^(3) = 4.18, *p* = .24) and diagnosis × segment (*χ*^2^(3) = 0.53, *p* = .91) interactions. There was no main effect of diagnosis (*χ*^2^(3) = 4.87, *p* = .18). These findings suggest that the risk group effect is not explained by diagnostic outcomes.

### 4.3 Face time and face foraging measures

#### 4.3.1 Risk group effects

Analyses were conducted using a similar model to that described above with the factors: risk group, time and trial segment. For face time proportion, the 3-way interaction of risk group × age × segment was not significant (*χ*^2^(1) = 1^.^18, *p* = .277) nor were the 2-way group × time (*χ*^2^(1) = 2^.^06, *p* = .151) and group × segment (*χ*^2^(1) = 0^.^97, *p* = .325) interactions. There was a significant main effect of group (*χ*^2^(1) = 4^.^85, *p* = .028) indicating that the at-risk group spent proportionally more time on the face relative to other AOIs. The 3-way interaction of group × time × segment was marginally significant (*χ*^2^(1) = 3.14, *p* = .077) and the 2-way interaction of group × segment (*χ*^2^(1) = 0.86, *p* = .353) was non-significant for group × time (*χ*^2^(1) = .67, *p* = .155). There was a significant main effect of group (*χ*^2^(1) = 8.41, *p* = .004). Taken together, these findings suggest that overall, infants at-risk are more likely to sample the face compared to other AOIs, and a trend toward this pattern being more pronounced at 14-months relative to 7-months.

#### 4.3.2 Diagnostic group effects

**Fig. 2:**
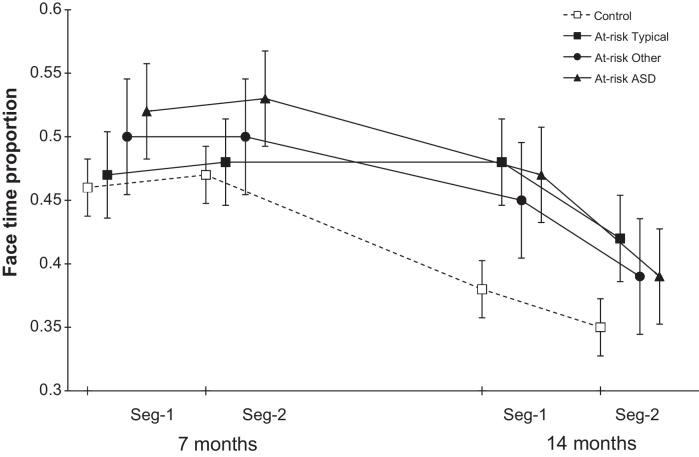
Face time: proportion of time spent on face relative to other AOIs.

**Fig. 3:**
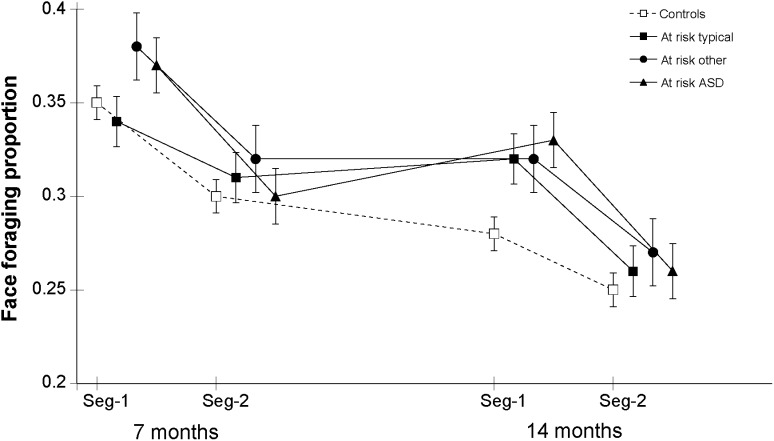
Face foraging: the ratio of sampling the face relative to all other sampled AOIs.

For face time proportion, estimated means for each diagnostic group are shown in Fig. 2. The 3-way interaction of diagnosis × age × segment was not significant (*χ*^2^(3) = 1.42, *p* = .70) nor were the 2-way diagnosis × time (*χ*^2^(3) = 2.81, *p* = .42) and diagnosis × segment (*χ*^2^(3) = 0.80, *p* = .85) interactions. There was no significant main effect of diagnosis (*χ*^2^(3) = 4.71, *p* = .19). For the face foraging proportion, the estimated means for each group are shown in Fig. 3. The 3-way interaction of group × time × segment was not significant (*χ*^2^(3) = 3.72, *p* = .29) nor were the 2-way interactions for group × time interaction (*χ*^2^(3) = 2.02, *p* = .57) and group × segment (*χ*^2^(3) = 4.53, *p* = .21). These findings suggest that the risk group effects are not related to diagnostic outcomes.

## 5 Discussion

In the current study, we addressed three complementary questions regarding the early development of the social brain in infants at-risk for autism. First, we tested a popular idea in developmental psychopathology positing that autism results from the lack of a bias to orient towards social information. Our findings suggest that, similar to other groups, infants who later develop ASD exhibit a clear face pop-out response, i.e., attentional capture by faces. Second, to examine the possible impact of atypical general mechanisms of attention, looking time and foraging measures provided complementary information regarding the distribution of infants’ looking behaviour beyond face pop out. Our findings indicate that, as a group, infants at-risk had a tendency to sample fewer stimuli in the array relative to the control group, with this being most clearly evident at the beginning of the second year and regardless of their clinical outcomes assessed at 3-years. Finally, we examined the interaction between social orienting and general attention mechanisms in the developing brain. Contrary to some current hypotheses, those infants who later developed autism were equally captured by faces relative to other groups at 7-months. In fact, infants with familial liability for autism as a group tended to be more captured by faces in this early developmental period. While these findings were marginal and require replication with other groups they appear to contradict social orienting and social reward models of autism, which argue for lesser engagement with people and faces early in life [20,13].

These findings are consistent with a growing body of evidence from less structured observational settings that orienting towards people does not distinguish, at least at six months of age, those infants who go on to a later diagnosis [53,68,69]. In the current study, we assessed the integrity of early developing neural systems biasing infants to orient and sustain attention to socially relevant stimuli. While the static arrays used in the current study may differ from the infants’ naturalistic environment, the task allowed us to isolate and directly test different factors contributing to previous findings based on observing infants in dynamic and naturalistic settings where they interact with their caregivers [68] or an experimenter [53,69].

Because autism is a complex condition encompassing symptoms in both social and non-social skills, others have suggested that in early infancy, multiple brain systems are likely to be affected, including those related to flexibly and efficiently allocating attention towards different stimuli in the environment [6]. Our findings support the growing consensus in research on infants at-risk that autism begins with subtle manifestations early in life which then transform over time into emergent symptoms in a minority of children. We found little evidence that autism-related symptoms are a consequence of early impairment of mechanisms that mediate attentional capture by socially-relevant information. However, mechanisms mediating efficient foraging of environmental stimuli in the presence of socially-relevant stimuli tended to distinguish infants at-risk as a group. Considering the two foraging measures together suggests that infants at-risk tend to only look at the face and a few other AOIs (while other infants distribute their looking more equally between AOIs), a finding possibly consistent with an emerging overly focal attention style.

Distribution of looking time and visual foraging are likely to reflect the combined operations of multiple sub-cortical and cortical systems giving rise to differences in response to social stimuli early on. This pattern suggests that interactions among multiple brain systems over the first two years give rise to variable pathways in infants at-risk. While it may appear paradoxical that those infants at-risk spent marginally more time on faces relative to other stimuli, the combination of typical face orienting mechanisms with differences in cortical mechanisms mediating efficient selection may manifest in a subset of infants at-risk looking longer towards faces.

The underlying mechanisms and the functional consequence of this increased proportional looking towards faces remain to be explored. Longer dwell time on faces or other visual stimuli have been previously associated with processing difficulties. For example, infants, who have a pattern of prolonged fixation time, rely more on local elements when processing visual stimuli [14,25,26]. Individual differences in looking time, i.e., ‘long vs. short lookers’ also predict later cognitive outcomes in typical infants [15] and ‘longer looking’ has been documented in other atypical populations such as preterm infants [57] or prenatal cocaine-exposed infants [17]. Specific effects of these attentional constraints on face processing have also been reported. ‘Long-lookers’ were better at detecting feature changes [14], potentially giving rise to differences in local vs. configural processing strategies [34]. Future studies should explore other measures of visual behaviour (e.g. fixation duration, scanning paths) to test the hypothesis of a visual processing deficit in infants who subsequently develop autism. Use of brain imaging methods such as EEG or fMRI will also support clarifying the underlying brain processes mediating the eye tracking findings observed in the current study.

The extent to which the behavioural patterns observed in the current study reflect processing differences that have functional consequences as development proceeds needs further investigation. In particular, the face pop-out measure in the current task is not sensitive to face properties which are believed to be important signals for social communication, like the up-right (vs. inverted) position or direct (vs. indirect) gaze. For example, typically developing six-month-old infants oriented equally frequently towards inverted faces and faces with averted gaze [28]. This orienting mechanism, while necessary, is not sufficient to appropriately modulate infants’ participation in social interactions.

Despite these limitations, our findings carry significant implications for ‘classical’ developmental accounts using autism as a model for understanding the emergence of the social brain. While it remains possible that autism results from early differences in response to socially-relevant information, our findings with a group of infants at-risk for autism decrease the likelihood that these early differences are primarily related to sub-cortical systems mediating an early bias to orient towards faces. This is in line with recent studies highlighting differences between adults with amygdala lesions and with ASD. ASD participants oriented to faces and eyes more often than amygdala patients but they did not modulate their orienting depending on task demands, an ability which probably depends on cortical functions (Birmingham, Cerf & Adolphs, 2011). Cortical mechanisms mediating efficient selection and distribution of attention appear to modulate infants’ early response to faces, reflecting interactions among multiple developing systems. A reasonable alternative to the classical accounts would be that autism provides a useful framework for understanding how differences in face processing mechanisms may emerge as a consequence of early atypical interactions among different systems. Future translational research concerned with developing early markers of autism can also benefit from the current findings through shifting the focus towards models of cumulative risk reflected in variable trajectories, a subset of which result in a diagnosis in toddlerhood or beyond [24].

## Figures and Tables

**Table 1:** Participant characteristics.

		Control	At-Risk			
			Combined	Typical	ASD	Other concerns^a^
Visit	Measure	Mean (sd) *n*	Mean (sd) *n*	Mean (sd) *n*	Mean (sd) *n*	Mean (sd) *n*
6–10 months	Age at visit (months)	7.4 (1.2) 50	7.3 (1.2) 54	7.1 (1.2) 24	7.5 (1.2) 17	7.3 (1.1) 12
	Mullen ELC SS	104.4 (11.3) 50	94.0 (12.8) 53	96.1 (11.8) 24	92.1 (17.3) 16	92.8 (8.1) 12
	Mullen NV T-score	56.2 (7.1) 50	51.5 (8.4) 53	52.6 (8.6) 24	49.9 (9.8) 16	51.3 (6.3) 12
	VABS ABC SS	101.8 (13.7) 49	92.1 (14.8) 53	95.7 (17.8) 23	90.0 (13.4) 17	87.6 (9.0) 12

12–15 months	Age at visit (months)	13.9 (1.3) 48	13.7 (1.6) 53	13.5 (1.7) 23	13.9 (1.6) 17	13.5 (1.2) 12
	Mullen ELC SS	106.1 (15.7) 47	97.4 (17.9) 53	103.3 (18.1) 23	89.2 (18.3) 17	99.8 (11.3) 12
	Mullen NV T-score	58.4 (8.3) 47	53.1 (10.3) 53	54.5 (10.7) 23	49.4 (10.9) 17	56.7 (6.3) 12
	VABS ABC SS	100.8 (8.9) 45	91.5 (13.8) 51	95.6 (10.3) 21	87.5 (13.7) 17	90.6 (18.5) 12

24-months	Age at visit (months)	23.9 (0.7) 47	23.9 (1.2) 52	23.9 (1.3) 24	24.0 (1.0) 16	23.8 (1.1) 12
	Mullen ELC SS	116.0 (14.0) 42	102.3 (19.8) 52	105.4 (17.5) 24	97.8 (24.7) 16	102.0 (16.8) 12
	Mullen NV T-score	56.9 (8.8) 43	51.6 (9.7) 52	53.7 (7.9) 24	49.4 (11.3) 16	50.2 (10.5) 12
	VABS ABC SS	108.2 (12.0) 47	101.5 (10.6) 52	103.5 (9.9) 24	100.0 (12.8) 16	99.3 (8.8) 12
	ADOS Communication		2.1 (1.6) 52	1.3 (1.2) 24	3.2 (1.8) 16	2.3 (1.3) 12
	ADOS Social		4.3 (3.0) 52	3.0 (2.8) 24	6.6 (2.9) 16	3.8 (1.6) 12
	ADOS Total		6.4 (4.3) 52	4.4 (3.8) 24	9.8 (4.3) 16	6.0 (2.1) 12

36-months	Age at visit (months)	38.2 (3.1) 48	37.7 (3.0) 53	38.1 (3.9) 24	37.8 (2.1) 17	36.7 (1.8) 12
	Mullen ELC SS	115.8 (16.3) 48	105.4 (21.5) 52	113.5 (13.3) 24	94.8 (28.5) 16	103.4 (19.0) 12
	Mullen NV T-score	57.8 (9.9) 48	52.6 (13.0) 52	57.1 (9.3) 24	45.3(15.8) 16	53.2 (12.1) 12
	VABS ABC SS	106.4 (9.1) 48	96.4 (12.2) 53	101.3 (8.7) 24	90.1 (14.6) 17	95.7 (10.8) 12
	ADOS Communication	2.5 (1.5) 48	3.3 (2.2) 53	2.0 (1.2) 24	4.2 (2.5) 17	4.8 (1.9) 12
	ADOS Social	3.2 (3.1) 48	4.9 (3.5) 53	2.0 (1.5) 24	7.4 (2.7) 17	7.3 (2.6) 12
	ADOS Total	5.6 (4.3) 48	8.3 (5.3) 53	4.0 (2.2) 24	11.7 (4.7) 17	12.1 (4.1) 12
	ADI Social		4.5 (5.3) 52	1.6 (1.7) 24	9.8 (5.5) 16	3.4 (4.9) 12
	ADI Communication		4.4 (4.8) 52	2.2 (1.8) 24	8.4 (5.1) 16	3.6 (5.5) 12
	ADI Beh/Rep Int		1.6 (2.0) 52	0.5 (0.9) 24	3.6 (2.2) 16	1.1 (1.3) 12

a Not meeting threshold for ASD diagnosis.

**Table 2:** The number of valid trials and proportion of trials with first look to the face in the face pop-out.

		7-month	14-months
Control	Valid trials (sd)	11.0 (2.8)	10.7 (3.0)
	% trials with first look to face (sd)	.48 (.15)	.53 (.18)
	*N*	50	47

At-risk combined	Valid trials (sd)	10.3 (2.4)	10.8 (3.0)
	% trials with first look to face (sd)	.52 (.20)	.57 (.23)
	*N*	51	52

At-risk typical	Valid trials (sd)	10.2 (2.5)	10.5 (2.8)
	% trials with first look to face (sd)	.54 (.21)	.61 (.20)
	*N*	21	22

At-risk other	Valid trials (sd)	10.3 (3.1)	11.8 (2.7)
	% trials with first look to face (sd)	.52 (.17)	.60 (.24)
	*N*	12	12

At-risk ASD	Valid trials (sd)	10.6 (1.7)	10.9 (3.2)
	% trials with first look to face (sd)	.49 (20)	.48 (.25)
	*N*	17	17

**Table 3:** Looking time and visual foraging measures.

			Control	At-risk			
				Combined	Typical	other	ASD
		*n*	46	51	21	12	17
Looking time	7 m	seg1	3.30(0.74)	3.17(0.74)	3.11(0.79)	2.98(0.74)	3.33(0.69)
		seg2	4.69(1.69)	4.35(1.60)	4.52(1.84)	3.67(0.90)	4.48(1.55)
	14 m	seg1	3.40(0.60)	3.34(0.60)	3.35(0.65)	3.33(0.60)	3.53(0.57)
		seg2	4.91(1.39)	5.00(1.58)	4.85(1.57)	5.10(1.44)	4.97(1.79)

Visual foraging	7 m	seg1	2.67(0.48)	2.66(0.55)	2.76(0.57)	2.57(0.61)	2.61(0.52)
		seg2	3.07(0.45)	3.01(0.65)	3.03(0.67)	2.84(0.71)	3.10(0.60)
	14 m	seg1	3.22(0.50)	2.96(0.56)	3.01(0.51)	2.98(0.53)	2.96(0.59)
		seg2	3.67(0.49)	3.41(0.59)	3.52(0.51)	3.45(0.53)	3.30(0.70)

Face time proportion	7 m	seg1	.46(.13)	.49(.17)	.47(.15)	.50(.19)	.52(.18)
		seg2	.47(.15)	.50(.17)	.48(.19)	.50(.17)	.53(.16)
	14 m	seg1	.38(.16)	.48(.18)	.48(.18)	.45(.19)	.47(.17)
		seg2	.35(.13)	.41(.16)	.42(.17)	.39(.11)	.39(.16)

Face foraging proportion	7 m	seg1	.35(.05)	.36(.09)	.34(.08)	.38(.10)	.37(.09)
		seg2	.30(.05)	.31(.06)	.31(.07)	.32(.05)	.30(.06)
	14 m	seg1	.28(.05)	.33(.08)	.32(.06)	.32(.07)	.33(.08)
		seg2	.25(.04)	.26(.06)	.26(.05)	.27(.03)	.26(.07)

## Footnotes

2 This software calculates the order in which areas of interest in each array would attract the attention of the participants, based on relative saliency. To calculate saliency it uses image properties known to attract fixations in visual scenes, like local contrast and orientation [35]. A winner takes all neural network calculates the point of highest density and draws the focus of attention to this area. For each object category we calculated how many times they would attract the first look across the 14 trials (Face 21%, Bird 28%, Mobile 21%, Face Noise 14%, Car 14%).
